# Facial Regioselective Synthesis of Novel Bioactive Spiropyrrolidine/Pyrrolizine-Oxindole Derivatives via a Three Components Reaction as Potential Antimicrobial Agents

**DOI:** 10.3390/molecules22030357

**Published:** 2017-02-26

**Authors:** Huwaida M. E. Hassaneen, Elshimaa M. Eid, Hamid A. Eid, Thoraya A. Farghaly, Yahia Nasser Mabkhot

**Affiliations:** 1 Chemistry Department, Faculty of Science, Cairo University, Giza 12613, Egypt; huwaidahassaneen@yahoo.com (H.M.E.H.); elshimaaeid80@hotmail.com (E.M.E.); profhamedead@gmail.com (H.A.E.); 2 Department of Chemistry, Faculty of Applied Science, Umm Al-Qura University, Makkah Almukkarramah 21514, Saudi Arabia; 3 Department of Chemistry, College of Science, King Saud University, P.O. Box 2455, Riyadh-11451, Saudi Arabia

**Keywords:** isatin, sarcosine, l-proline, azomethineylide, 1,3-dipolar cycloaddition, antimicrobial activity

## Abstract

This article presents the synthesis of new derivatives of spirooxindole-spiropiperidinone- pyrrolidines **6a**–**j** and spirooxindole-spiropiperidinone-pyrrolizines **8a**–**j,** through a 1,3-dipolar cycloaddition reaction of azomethineylides generated from isatin, sarcosine, and l-proline, through a decarboxylative route with dipolarophile **4a**–**j**. All of the newly synthesized compounds were evaluated for their antimicrobial activities and their minimum inhibitory concentration (MIC) against most of the test organisms. The tested compounds displayed excellent activity against all of the tested microorganisms.

## 1. Introduction

In recent years, multicomponent reactions (MCR’s) [1,2,3] leading to interesting heterocyclic scaffolds have emerged as powerful tools for the diverse needs in combinatorial approaches for the synthesis of bioactive compounds, creating distinct chemical libraries of drug-like molecules for biological screening [4,5]. Spiro compounds represent an important class of naturally occurring substances with highly pronounced biological properties [6,7]. Spirooxindole ring systems are found in a number of alkaloids, such as horsifiline, spirotryprostain, and (+) elacomine [8], and are used in biological applications as antimicrobial and antitumor agents and as inhibitors of the human NKI receptor [9]. Additionally, spirooxindole rings containing the pyrrolidine and pyrrolizine ring system are found in various natural products as fundamental nuclei and are well recognized for exhibiting a wide range of pharmacological and biochemical behaviors [10,11,12]. The 1,3-cycloaddition methodology is one of the simplest tools for the construction of five-membered heterocycles. The ease of generating a 1,3-dipolar, coupled with the observed highly regio- and stereoselective nature of cycloaddition, has led to a number of syntheses that utilize such a reaction [13]. Combining several transformations into a one-pot reaction has proved to be an excellent strategy for increasing the efficiency of organic synthesis [14]. In particular, azomethineylides have been used to synthesize pyrrolidines and pyrrolizines with various substitutions, allowing the introduction of several functional groups in a single operation [15]. As part of our own interest in the synthesis of biologically active heterocyclic compounds [16,17,18,19,20,21,22,23], we report an efficient methodology for the synthesis of novel spirooxindole-spiropiperidinone-pyrrolidine and spirooxindole-spiropiperidinone-pyrrolizine derivatives, and investigate their antimicrobial activities.

## 2. Results and Discussion

### 2.1. Chemistry

**Scheme 1:**
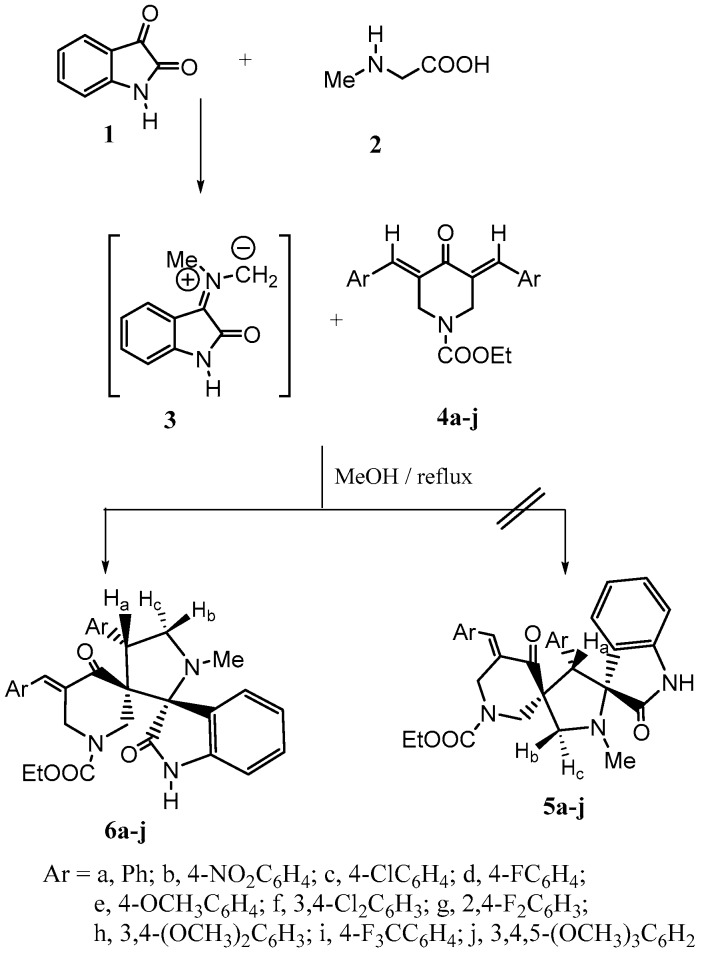
Synthesis of spiropyrrolidine bisoxindoles.

In our initial endeavor, the Knoevenagel adduct **4** was prepared via condensation of 1-ethoxycarbonyl-4-piperidinone with aromatic aldehydes, in the presence of a base catalyst [24]. Then, the three-component reaction of isatin **1**, sarcosine **2**, and ethyl 3,5-*bis*[phenylmethylidene]-4-oxopiperidine-*N*-carboxylate **4a**, as a simple model substrate, was investigated, to establish the feasibility of the strategy and optimize the reaction conditions. The reaction was carried out at 60 °C in methanol and took around 120 min. It was then cooled to room temperature and the solid that formed was filtered and recrystallized from methanol to furnish the functional dispiropyrrolidinyloxindole in a highly regioselective manner, to afford **6a** (Scheme 1).

The dispiroheterocyclic ring structure of products **6a**–**j** was confirmed by spectroscopic data (IR, ^1^H- and ^13^C-NMR) and elemental analysis. The IR spectrum of **6a** revealed the presence of a carbonyl stretching vibration band at 1685 cm^−1^, showing an increase of 18 cm^−1^ from the normal value observed for 3,5-*bis*[phenylmethylidene]-4-oxipiperidine-*N*-carboxylate **4a**, indicating the loss of conjugation from one side. It also exhibited two bands at 1718 and 1710 cm^−1^, due to the carbonyl group of oxindole and ester moiety, respectively. On the other hand, the ^1^H-NMR spectrum of **6a** showed a sharp singlet signal at δ 1.98 for thepyrroline-N-CH_3_ proton. The benzylic proton H_a_ exhibited a doublet of the doublet in the region δ 4.82, with (*J* = 10.7, 9.0 Hz). The H_c_ and H_b_ protons appeared as a doublet of the doublet in the region δ 3.91 with (*J* = 10.7, 7.0 Hz) and 3.43 with (*J* = 9.0, 7.0 Hz), respectively. The protons of the piperidinone ring appeared as a multiple signal in the region δ 3.32–3.39. Also, the olefinic proton was observed at δ 7.59 as a singlet, whereas the aromatic protons appeared as multiplets in the region δ 6.75–7.47, and as a broad singlet at δ 8.56 for the NH proton of the oxindole ring.

The regiochemistry of the product **6a** was confirmed by the ^1^H-NMR spectra. The benzylic proton H_a_ was observed at δ 4.82, as a doublet of the doublet. If the other isomer was formed, one would expect a singlet, instead of a doublet of the doublet, for this benzylic proton. The ^13^C-NMR spectra of **6a** exhibited peaks at δ 64.2 ppm and 76.2 ppm for two spiro carbons. The peaks at δ 154.32, δ 173.2, and at δ 202.12 ppm, were due to the ester carbonyl, oxindole carbonyl, and keto carbonyl carbons of the N-COOEt piperidinone ring system, respectively. The mass spectra of **6a** showed a molecular ion peak at *m*/*z* 521 (M^+^), which further confirmed the formation of a mono-adduct.

After the formation of the mono-adduct, the reaction failed to give the *bis*-adduct, even with an excess of 1,3-dipole and a prolonged reaction time. This may be due to the steric hindrance and fixing of the geometry of the spiropyrrolidine ring, which prevents afurther attack of 1,3-dipole on the other exocyclic double bond. However, *bis*-adducts are formed if a small 1,3-dipole is generated [25].

This reaction proceeds through the decarboxylative condensation of isatin **1** with sarcosine **2**, to generate an azomethineylide **3**. The generated 1,3-dipole cycloaddition with the dipolarophile **4a**–**j** produces novel dispiro-oxindolopyrrolidines **6a**–**j** (Scheme 1).

To enhance the yield, endeavors were made to streamline other response parameters, including the solvents and reaction temperature. Thus, the reaction was studied in different solvents that included THF, toluene, CH_3_CN, CH_3_OH, EtOH, and H_2_O (Table 1, entries 1–6). To our satisfaction, the reaction in methanol led to the desired product with an almost quantitative yield (92%) (Table 1, entry 4), while ethanol as a solvent produced the product with only an 85% yield (Table 1, entry 5). In general, the reactions carried out in protic solvents yielded better results than those in aprotic solvents. However, when water was employed as the solvent, no product was detected (Table 1, entry 6). This might be caused by the poor solubility of isatin **1** and ethyl 3,5-*bis*[phenylmethylidene]-4-oxopiperidine-*N*-carboxylate **4a**–**j** in water.

The temperature influenced the rate of the reaction. Reducing the reaction temperature resulted in low reactivity (Table 1, entries 7 and 8), while elevating the temperature to 60 °C provided the best results. Based on the comprehensive consideration of the reaction temperature and yield, the optimal reaction conditions were established, as shown in Table 1 entry 4.

Considering the optimized conditions, the 1,3-dipolar cycloaddition reaction of Knoevenagel adducts with different structures, was investigated. As shown in Table 2, a variety of Knoevenagel adducts proved to be excellent dipolarophiles for this reaction and provided the corresponding spiropyrrolidine bisoxindoles with good yields (up to 95%) (Table 2, entries 1–10).

Substituents on the aryl groups lightly influenced the yields. Generally, Knoevenagel adducts with electron-donating groups produced lower yields than those with electron-withdrawing groups (Table 2).

**Scheme 2:**
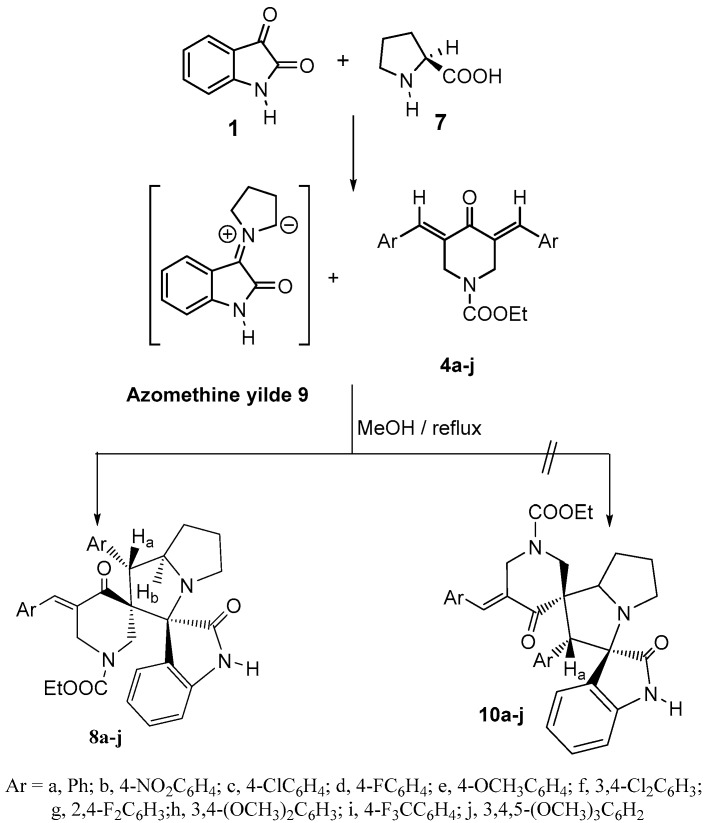
Synthesis of spiropyrrolizinebisoxindoles.

On the basis of the above mentioned results, we extended our protocol to the synthesis of dispiro-oxindolopyrrolizines **8a**–**j** from isatin **1**, aminoacid **7** such as l-proline, and ethyl 3,5-*bis*[arylmethylidene]-4-oxipiperidine-*N*-carboxylate **4a**–**j** in methanol, to yield a single product, as evidenced by TLC and spectral analysis (Scheme 2).

For example, in the ^1^H-NMR spectra of **8a**, the H_a_ appears as a doublet at δ 3.26 ppm (*J* = 10.2 Hz) and multiplet of H_b_ at δ 2.51–2.55 ppm. The singlet at δ 8.53 ppm is due to the NH of the oxindole ring. The ^13^C-NMR spectra of **8a** exhibited peaks at δ 62.1 ppm and 71.4 ppm for two spiro carbons. The peaks at δ 154.5, δ 173.2, and δ 199.2 ppm were due to the ester carbonyl, oxindole carbonyl, and keto carbonyl carbons of the N-COOEt piperidinone ring system, respectively. The mass spectra of **8a** showed a molecular ion peak at *m*/*z* 547 (M^+^), which further confirmed the formation of a mono-adduct.

Several advantages, such as a high yield, and simple experimental and isolation procedures, render the methanol an efficient route for the synthesis of spiro-frameworks from isatin, sarcosine, and l-proline, that are important compounds in organic and medicinal chemistry.

### 2.2. Antimicrobial Activity

The in vitro antimicrobial screening of compounds prepared in this study was carried out using the cultures of four bacteria species, namely, the Gram positive bacteria, *Streptococcus pneumonia* (RCMB 010010) (SP) and *Bacillis subtilis* (RCMB 010067) (BS), and the Gram negative bacteria, *Pseudomonas aeruginosa* (RCMB 010043) (PS), and *Escherichia coli* (RCMB 010052) (EC); as well as four fungal strains, including *Aspergillus fumigates* (RCMB 02568) (AF), *Syncephalastrumracemosum* (RCMB 05922) (SR), *Geotricumcandidum* (RCMB 05097) (GC), and *Candida albicans* (RCMB05036) (CA). Amphotericin B as an antifungal agent, Ampicillin as an antibacterial agent for gram (+) bacteria, and Gentamicin as an antibacterial agent for gram (−) bacteria, were used as references to evaluate the potency of the tested compounds under the same conditions. Most of the newly synthesized compounds showed excellent results with respect to the control drugs. The results of antimicrobial activities are shown in Table 3. The data in Table 3 revealed that most of the compounds have a superior and significant antibacterial potency to antifungal activity. It is clear from the data in Table 3 that compounds **8b**, **8e**, **8g** and **6c** exhibited the highest potency against tested organisms, with respect to the reference drugs. The other derivatives showed moderate activity against the microorganisms used. The compounds **6c** and **8g** exhibited antimicrobial activity against all of the tested microorganisms. It has also been observed that the newly synthesized compounds exhibited more promising antibacterial activity against Gram positive bacteria than Gram negative bacteria, displaying the highest activity against *Bacillis subtilis* (RCMB 010067).

The minimum inhibitory concentration (MIC) of the most active synthesized compounds against highly inhibited organisms, is reported in Table 4. Compounds **8b**, **8e**, **8g** and **6c** revealed the lowest MICs (0.06, 0.015, 0.015, and 0.015 µg/mL) against *Bacillis subtilis* (RCMB 010067), respectively.

The inhibitory concentration of the most active synthesized compounds against 50% of microorganism growth (IC_50_), is reported in Table 5. Compounds **8b**, **8e**, **8g** and **6c** revealed the lowest IC_50_ values (2.92, 1.34, 1.36, and 1.24 µg/mL) against *Bacillis subtilis* (RCMB 010067), respectively. 

Then, a study of the structure-activity relationships (SAR) showed that **8** compounds with a phenyl-hexahydro-1*H*-pyrrolizine ring, were more capable of improving the antimicrobial activity than compounds **6** with (-CH_3_) a pyrrolidine ring. 

According to the antimicrobial data, we evaluated the effect of introducing different substituent group(s) at phenyl rings in synthesized compounds. The introduction of electron-withdrawing groups (halogen groups or -NO_2_) showed better antimicrobial effects than the electron-donating group (4-OCH_3_, 3,4,5-(OCH_3_)_3_).

## 3. Experimental Section

### 3.1. General Procedures

Melting points were determined using a Gallenkamp electro-thermal apparatus and wereuncorrected. IR spectra were recorded as KBr disc, using a Shimadzu FTIR-prestige 21 spectrophotometer. **^1^**H- and ^13^C-NMR spectra were recorded in DMSO-*d*_6_ as solvents (at 300 MHz for ^1^H and 75 MHz for ^13^C) on a Varian Mercury NMR spectrometer, using TMS as the internal standard. Chemical shifts δ are reported in parts per million units (ppm), and *J* values are given in hertz. The mass spectra were recorded on a GCeMS-QP1000 EX mass spectrometer at 70 eV. Elemental analyzes were measured using a German made elementary vario LIII CHNS analyzer. Antibacterial activity was studied at the Regional Center for Mycology and Biotechnology at Al-Azhar University, Cairo, Egypt. Compounds **4a**–**c**, **4e** and **4h** were prepared as previously reported in the respective literature [24].

### 3.2. General Procedure for the Preparation of 3,5-bis[Arylmethylidene]-4-oxopiperidine-N-carboxylate *(**4d**,**f**,**g**,**i**,**j**)*

A mixture of 1-ethoxycarbonyl-4-piperidinone (1.71 mL, 10 mmol) and aromatic aldehydes (20 mmol) in methanol (20 mL), in the presence of potassium hydroxide, was used as the base catalyst (1 g). The mixture was stirred at room temperature for 30 min. The solid formed was collected, washed with methanol, and crystallized from proper solvent, to give the compounds, as listed below:

*Ethyl 3,5-bis[4-fluorophenyl-methylidene]-4-oxopiperidine-N-carboxylate* (**4d**). Yellow solid, m.p. 210–212 °C (EtOH); IR (KBr): 1717, 1687 (2CO) cm^−1^. ^1^H-NMR (DMSO-*d*_6_); 1.12 (t, 3H, *J* = 7.2 Hz, CH_3_ of CH_2_CH_3_), 3.92 (s, 4H, 2CH_2_ of piperidinone), 4.75 (q, 2H, *J* = 7.2 Hz, CH_2_ of CH_2_CH_3_), 7.40–7.55 (m, 8H, Ar-H), 7.67 (s, 2H, 2CH=). ^13^C-NMR (DMSO-*d*_6_); 13.2, 59.5, 45.5, 117.0, 128.2, 133.2, 142.3, 145.9, 164.2, 155.3, 185.1. MS *m/z* (%): 385 (M^+^ + 2, 40), 384 (M^+^ + 1, 23), 383 (M^+^, 45), 206 (14), 97 (30), 57 (15). Anal. for C_22_H_19_F_2_NO_3_ (383.39): calcd. C, 68.92; H, 5.0; N, 3.65. Found C, 68.65; H, 5.10; N, 3.54.

*Ethyl 3,5-bis[3,4-dichlorophenyl-methylidene]-4-oxopiperidine-N-carboxylate* (**4f**). Yellow solid, m.p. 208–210 °C (EtOH/Dioxane); IR (KBr): 1720, 1688 (2CO) cm^−1^. ^1^H-NMR (DMSO-*d*_6_): δ 1.13 (t, 3H, *J* = 7 Hz, CH_3_ of CH_2_CH_3_), 3.93 (s, 4H, 2CH_2_ of piperidinone), 4.73 (q, 2H, *J* = 7 Hz, CH_2_ of CH_2_CH_3_), 7.45–7.56 (m, 6H, Ar-H), 7.66 (s, 2H, 2CH=). MS *m/z* (%): 487 (M^+^ + 2, 50), 486 (M^+^ + 1, 30), 485 (M^+^, 32), 167 (15), 147 (10). Anal. For C_22_H_17_Cl_4_NO_3_ (485.18): calcd. C, 54.46; H, 3.54; N, 2.89. Found: C, 54.45; H, 3.82; N, 2.63.

*Ethyl 3,5-bis[2,4-difluorophenyl-methylidene]-4-oxopiperidine-N-carboxylate* (**4g**). Yellow solid, m.p. 167–168 °C (EtOH/Dioxane); IR (KBr): 1715, 1686 (2CO) cm^−1^. ^1^H-NMR (DMSO-*d*_6_): δ 1.2 (t, 3H, *J* = 7.2 Hz, CH_3_ of CH_2_CH_3_), 3.91 (s, 4H, 2CH_2_ of piperidinone), 4.56 (q, 2H, *J* = 7.2 Hz, CH_2_ of CH_2_CH_3_), 7.53–7.76 (m, 6H, Ar-H), 7.84 (s, 2H, 2CH=). ^13^C-NMR (DMSO-*d*_6_): δ 14.22, 59.3, 46.7, 105, 112.2, 119.2, 130.4, 140.2, 145.9, 154.7, 160.3, 165.1, 185.8. MS *m/z* (%): 421 (M^+^ + 2, 5), 420 (M^+^ + 1, 23), 419 (M^+^, 42), 206 (12), 97 (10), 57 (9). Anal. for C_22_H_17_F_4_NO_3_ (419.38): calcd. C, 63.00; H, 4.27; N, 3.62. Found: C, 63.11; H, 4.26; N, 3.42.

*Ethyl 3,5-bis[4-trifluoromethylphenyl-methylidene]-4-oxo-piperidine-N-carboxylate* (**4i**). Yellow solid, m.p. 147–149 °C (EtOH/Dioxane); IR (KBr): 1709, 1691 (2CO) cm^−1^. ^1^H-NMR (DMSO-*d*_6_): δ 1.19 (t, 3H, *J* = 7.1 Hz, CH_3_ of CH_2_CH_3_), 3.86 (s, 4H, 2CH_2_ of piperidinone), 4.24 (q, 2H, *J* = 7.1 Hz, CH_2_ of CH_2_CH_3_), 7.21–7.53 (m, 8H, Ar-H), 7.83 (s, 2H, 2CH=). MS *m/z* (%): 485 (M^+^ + 2, 25), 484 (M^+^ + 1, 38), 483 (M^+^, 62), 158 (23), 145 (20). Anal. for C_24_H_19_F_6_NO_3_ (483.41): calcd. C, 59.63; H, 3.96; N, 2.90. Found: C, 59.91; H, 4.00; N, 2.71.

*Ethyl 3,5-bis[3,4,5-trimethoxyphenyl-methylidene]-4-oxopiperidine-N-carboxylate* (**4j**). Yellow solid, m.p. 126–127 °C (EtOH); IR (KBr): 1718, 1686 (2CO) cm^−1^. ^1^H-NMR (DMSO-*d*_6_): δ 1.2 (t, 3H, *J* = 7.2 Hz, CH_3_ of CH_2_CH_3_), 2.49 (s, 4H, 2CH_2_ of piperidinone), 3.17 (s, 6H, CH_3_ of OCH_3_), 3.65 (s, 6H, CH_3_ of OCH_3_), 3.72 (s, 6H, CH_3_ of OCH_3_), 4.8 (q, 2H, *J* = 7.2 Hz, CH_2_ of CH_2_CH_3_), 6.84 (s, 4H, Ar-H), 7.24 (s, 2H, 2CH=). ^13^C-NMR (DMSO-*d*_6_): δ 13.6, 58.9, 46.4, 56.1, 56.5, 105, 129.2, 140.1, 151.2, 140.5, 145.7, 154.9, 186.7. MS *m/z* (%): 529 (M^+^ + 2, 6), 528 (M^+^ + 1, 20), 527 (M^+^, 63), 97 (15), 57 (9). Anal. for C_28_H_33_NO_9_ (527.57): calcd. C, 63.75; H, 6.31; N, 2.66. Found: C, 63.50; H, 6.41; N, 2.69.

### 3.3. General Synthesis of Dispiropyrrolidine Oxindole Derivatives *(**6a**–**j**)*

A reaction mixture of isatin **1** (1.47 g, 10 mmol), sarcosine **2** (0.89 g, 10 mmol), and Ethyl 3,5-*bis*(arylmethylidene)-*N*-carboxyl-4-piperidinone **4a**–**j** (10 mmol), was produced by refluxing it in methanol for 120 min at 60 °C, and was then poured on water. The solid formed was collected and crystallized from a suitable solvent to produce white to brown crystals of compounds (**6a**–**j**).

*Ethyl 1-N-methyl-spiro[2.3′]oxindole-spiro[3.3′′]5′′-benzylidene-1′′-N-carboxylate-4′′-piperidinone-4-phenyl-pyrrolidine* (**6a**). White crystals, m.p. 160–162 °C (EtOH/Dioxane); IR (KBr): 3253 (NH), 1718, 1710, 1685 (3CO) cm^−1^. ^1^H-NMR (DMSO-*d*_6_): δ 1.15 (t, 3H, *J* = 7.2 Hz, CH_3_ of CH_2_CH_3_), 1.98 (s, 3H, NCH_3_), 3.32, 3.39 (2s, 4H, 2CH_2_ of piperidinone ring), 3.43 (dd, *J* = 9.0, 7.0 Hz, 1H_b_), 3.91 (dd, *J* = 10.7, 7.0 Hz, 1H_c_), 4.39 (q, 2H, *J* = 7.2 Hz, CH_2_ of CH_2_CH_3_), 4.82 (dd, *J* = 10.7, 9.0 Hz, 1H_a_), 6.75–7.47 (m, 14H, Ar-H), 7.59 (s, 1H, CH=), 8.56 (s, 1H, D_2_O-exchangeable, NH). ^13^C-NMR (DMSO-*d*_6_): δ 13.7, 29.9, 38.1, 43.2, 46.7, 55.3, 59.3, 64.2, 76.2, 121.6, 124.1, 126.3, 126.4, 126.5, 127.4, 127.9, 128.2, 128.5, 136.2, 136.4, 138.2, 139.5, 141.2, 153.9, 154.3, 173.2, 202.1. MS *m*/*z* (%): 523 (M^+^ + 2, 9), 522 (M^+^ + 1, 25), 521 (M^+^, 35), 130 (14), 77 (30). Anal. for C_32_H_31_N_3_O_4_ (521.62): calcd. C, 73.68; H, 5.99; N, 8.06.Found C, 73.53; H, 5.94; N, 8.02.

*Ethyl 1-N-methyl-spiro[2.3′]oxindole-spiro[3.3′′]5′′-(4-nitro)benzylisdene-1′′-N-carboxylate-4′′-piperidinone-4-(4-nitro)phenyl-pyrrolidine* (**6b**). Yellow crystals, m.p. 175–176 °C (Dioxane); IR (KBr): 3262 (NH), 1714, 1701, 1692(3CO) cm^−1^. ^1^H-NMR (DMSO-*d*_6_): δ 1.18 (t, 3H, *J* = 7 Hz, CH_3_ of CH_2_CH_3_), 2.03 (s, 3H, NCH_3_), 3.22 (dd, *J* = 9.0, 7.0 Hz, 1H_b_), 3.30, 3.45 (2s, 4H, 2CH_2_ of piperidinone ring), 3.84 (dd, *J* = 10.7, 7.0 Hz, 1H_c_), 4.18 (q, 2H, *J* = 7 Hz, CH_2_ of CH_2_CH_3_), 4.79 (dd, *J* = 10.7, 9.0 Hz, 1H_a_), 6.76–7.82 (m, 12H, Ar-H), 8.40 (s, 1H, CH=), 11.01 (s, 1H, D_2_O-exchangeable, NH). ^13^C-NMR (DMSO-*d*_6_): δ 13.5, 29.9, 37.8, 43.5, 46.9, 55.8, 58.9, 64.3, 76.5, 121.6, 124.1, 126.1, 126.4, 126.6, 127.1, 127.4, 128.6, 128.8, 128.9, 136.2 , 136.3, 138.2, 139.5, 141.5, 153.9, 158.3, 175.2, 201.5. MS *m/z* (%): 613 (M^+^ + 2, 8), 612 (M^+^ + 1, 35), 611 (M^+^, 98), 242 (12), 205 (15), 97 (15). Anal. For C_32_H_29_N_5_O_8_ (611.61): calcd. C, 62.84; H, 4.78; N, 11.45. Found: C, 62.78; H, 4.69; N, 11.36.

*Ethyl 1-N-methyl-spiro[2.3′]oxindole-spiro[3.3′′]5′′-(4-chloro)benzylidene-1′′-N-carboxylate-4′′-piperidinone-4-(4-chloro)phenyl-pyrrolidine* (**6c**). Yellow crystals, m.p. 185–187 °C (EtOH/Dioxane); IR (KBr): 3251 (NH), 1716, 1708, 1686 (3CO) cm^−1^. ^1^H-NMR (DMSO-*d*_6_): δ 1.16 (t, 3H, *J* = 7.2 Hz, CH_3_ of CH_2_CH_3_), 1.97 (s, 3H, NCH_3_), 3.11 (dd, *J* = 9.0, 7.0 Hz, 1H_b_), 3.29, 3.38 (2s, 4H, 2CH_2_ of piperidinone ring), 3.89 (dd, *J* = 10.7, 7.0 Hz, 1H_c_), 4.20 (q, 2H, *J* = 7.2 Hz, CH_2_ of CH_2_CH_3_), 4.72 (dd, *J* = 10.7, 9.0 Hz, 1H_a_), 6.72–7.49 (m, 12H, Ar-H), 7.56 (s, 1H, CH=), 10.48 (s, 1H, D_2_O-exchangeable, NH). ^13^C-NMR (DMSO-*d*_6_): δ 13.8, 29.8, 37.8, 43.2, 46.6, 55.5, 59.1, 63.9, 76.1, 121.2, 124.1, 126.4, 127.2,128.4, 128.6, 128.8, 130.1, 131.1, 133.4, 133.8, 136.6 , 137.6, 139.3, 139.7, 152.6, 154.0, 172.3, 203.9. MS *m/z* (%): 592 (M^+^ + 2, 6), 591 (M^+^ + 1, 34), 590 (M^+^, 88), 242 (12), 97 (15). Anal. For C_32_H_29_Cl_2_N_3_O_4_ (589.50): calcd. C, 65.09; H, 4.95; N, 7.12.Found C, 65.01; H, 4.86; N, 7.06.

*Ethyl 1-N-methyl-spiro[2.3′]oxindole-spiro[3.3′′]5′′-(4-fluoro) benzylidene-1′′-N-carboxylate-4′′-piperidinone-4-(4-fluoro)phenyl-pyrrolidine* (**6d**). Brown crystals, m.p. 215–217 °C (Dioxane); IR (KBr): 3256 (NH), 1720, 1711, 1684 (3CO) cm^−1^. ^1^H-NMR (DMSO-*d*_6_): δ 1.19 (t, 3H, *J* = 7.4 Hz, CH_3_ of CH_2_CH_3_), 2.18 (s, 3H, NCH_3_), 3.17 (dd, *J* = 9.0, 7.0 Hz, 1H_b_), 3.36, 3.48 (2s, 4H, 2CH_2_of piperidinone ring), 3.86 (dd, *J* = 10.5, 7.0 Hz, 1H_c_), 4.19 (q, 2H, *J* = 7.4 Hz, CH_2_ of CH_2_CH_3_), 4.71 (dd, *J* = 10.5, 9.0 Hz, 1H_a_), 6.75–7.33 (m, 12H, Ar-H), 7.40 (s, 1H, CH=), 10.5 (s, 1H, D_2_O-exchangeable, NH).^13^C-NMR (DMSO-*d*_6_): δ 13.5, 29.7, 37.9, 42.9, 46.6, 54.7, 59.1, 63.7, 76.1, 114.7 ,115.3, 121.4, 124.3, 126.2, 128.1, 128.5, 130.2, 130.9, 136.4, 136.7, 137.9, 139.5, 159.8, 162.2, 152.6, 154.4, 173.4, 201.6. MS *m/z* (%): 559 (M^+^ + 2, 3), 558 (M^+^ + 1, 35), 557 (M^+^, 85), 206 (10), 97 (8). Anal for C_32_H_29_F_2_N_3_O_4_ (557.60): calcd. C, 68.93; H, 5.24; N, 7.54.Found C, 68.87; H, 5.21; N, 7.49.

*Ethyl 1-N-methyl-spiro[2.3′]oxindole-spiro[3.3′′]5′′-(4-methoxy) benzylidene-1′′-N-carboxylate-4′′-piperidinone-4-(4-methoxy)phenyl-pyrrolidine* (**6e**). Yellow crystals, m.p. 118–120 °C (Methanol); IR (cm^−1^): 3251 (NH), 1716, 1702, 1674 (3CO) cm^−1^. ^1^H-NMR (DMSO-*d*_6_): δ 1.13 (t, 3H, *J* = 7.2 Hz, CH_3_ of CH_2_CH_3_), 2.09 (s, 3H, NCH_3_), 3.25 (dd, *J* = 9.0, 7.0 Hz, 1H_b_), 3.33, 3.49 (2s, 4H, 2CH_2_ of piperidinone ring), 3.85, 3.87 (2s, 6H, 2OCH_3_), 3.89 (dd, *J* = 10.8, 7.0 Hz, 1H_c_), 4.22 (q, 2H, *J* = 7.2 Hz, CH_2_ of CH_2_CH_3_), 4.79 (dd, *J* = 10.8, 9.0 Hz, 1H_a_), 6.72–7.59 (m, 12H, Ar-H), 7.65 (s, 1H, CH=), 10.41 (s, 1H, D_2_O-exchangeable, NH). ^13^C-NMR (DMSO-*d*_6_): δ 13.9, 29.6, 38.0, 43.4, 46.9, 54.9, 55.9, 56.2, 59.3, 64.2, 76.2, 113.4, 114.1, 121.3, 124.2, 126.3, 127.3, 128.4, 129.4, 129.6, 133.4, 136.1, 138.3, 139.6, 152.8, 154.2, 157.6, 159.8, 172.0, 202.5. MS *m/z* (%): 583 (M^+^ + 2, 4), 582 (M^+^ + 1, 35), 581 (M^+^, 90), 244 (10), 206 (8), 95 (19). Anal. For C_34_H_35_N_3_O_6_ (581.67): calcd. C, 70.21; H, 6.07; N, 7.22. Found C, 70.19; H, 6.10; N, 7.20.

*Ethyl 1-N-methyl-spiro[2.3′]oxindole-spiro[3.3′′]5′′-(3,4-dichloro)benzylidene-1′′-N-carboxylate-4′′- piperidinone-4-(3,4 dichloro)phenyl-pyrrolidine* (**6f**). Yellow crystals, m.p. 213–214 °C (Dioxane);; IR (cm^−1^): 3400 (NH), 1715, 1713, 1692 (3CO) cm^−1^. ^1^H-NMR (DMSO-*d*_6_): δ 1.12 (t, 3H, *J* = 7.1 Hz, CH_3_ of CH_2_CH_3_), 1.98 (s, 3H, NCH_3_), 3.18 (dd, *J* = 9.1, 7.0 Hz, 1H_b_), 3.30, 3.45 (2s, 4H, 2CH_2_ of piperidinone ring), 3.88 (dd, *J* = 10.6, 7.0 Hz, 1H_c_), 4.28 (q, 2H, *J* = 7.1 Hz, CH_2_ of CH_2_CH_3_), 4.73 (dd, *J* = 10.6, 9.1 Hz, 1H_a_), 6.80–7.65 (m, 10H, Ar-H), 7.66 (s, 1H, CH=), 10.4 (s, 1H, D_2_O-exchangeable, NH). ^13^C-NMR (DMSO-*d*_6_): δ 13.6, 29.2, 38.2, 42.9, 46.7, 54.7, 59.3, 63.2, 76.1, 121.6, 124.1, 126.4, 127.9, 129.5, 129.8, 128.9, 130.2, 130.3, 136.1, 132.5, 132.6, 133.4, 134.8, 137.8, 139.5, 140.6, 153.9, 154.5, 173.1, 202.8. MS *m/z* (%): 661 (M^+^ + 2, 48), 660 (M^+^ + 1, 34.5), 659 (M^+^, 89), 657 (70), 357 (10), 339 (20), 338 (5), 145 (5). Anal. For C_32_H_27_Cl_4_N_3_O_4_ (659.38): calcd. C, 58.29; H, 4.13; N, 6.37.Found C, 58.28; H, 4.11; N, 6.36.

*Ethyl 1-N-methyl-spiro[2.3′]oxindole-spiro[3.3′′]5′′-(2,4-difluoro)-benzylidene-1′′-N-carboxylate-4′′-piperidinone-4-(2,4-difluoro)phenyl-pyrrolidine* (**6g**). Yellow crystals, m.p. 130–132 °C (EtOH/Dioxane); IR: 3255 (NH), 1716, 1692, 1660 (3CO) cm^−1^. ^1^H-NMR (DMSO-*d*_6_): δ 1.15 (t, 3H, *J* = 7.2 Hz, CH_3_ of CH_2_CH_3_), 1.98 (s, 3H, NCH_3_), 3.29 (dd, *J* = 9.0, 7.0 Hz, 1H_b_), 3.34, 3.46 (2s, 4H, 2CH_2_ of piperidinone ring), 3.82 (dd, *J* = 10.7, 7.0 Hz, 1H_c_), 4.29 (q, 2H, *J* = 7.2 Hz, CH_2_ of CH_2_CH_3_), 4.73 (dd, *J* = 10.7, 9.0 Hz, 1H_a_), 6.73–7.21 (m, 10H, Ar-H), 7.52 (s, 1H, CH=), 10.52 (br., 1H, D_2_O-exchangeable, NH). MS *m/z* (%): 595 (M^+^ + 2, 6), 594 (M^+^ + 1, 35), 593 (M^+^, 78), 339 (20), 325 (8), 306 (7), 113 (5). Anal. For C_32_H_27_F_4_N_3_O_4_ (593.58): calcd. C, 64.75; H, 4.59; N, 7.08. Found: C, 64.69; H, 4.50; N, 7.02.

*Ethyl 1-N-methyl-spiro[2.3′]oxindole-spiro[3.3′′]5′′-(3,4-dimethoxy)benzylidene-1′′-N-carboxylate-4′′-piperidinone-4-(3,4-dimethoxy)phenyl-pyrrolidine* (**6h**). Brown crystals, m.p. 95–98 °C (Methanol); IR: 3260 (NH), 1712, 1695, 1659 (3CO) cm^−1^. ^1^H-NMR (DMSO-*d*_6_); δ 1.18 (t, 3H, *J* = 6.9 Hz, CH_3_ of CH_2_CH_3_), 2.04 (s, 3H, NCH_3_), 3.23 (dd, *J* = 9.0, 7.0 Hz, 1H_b_), 3.33, 3.47 (2s, 4H, 2CH_2_of piperidinone ring), 3.67, 3.69 (2s, 6H, 2OCH_3_), 3.79, 3.82 (2s, 6H, 2OCH_3_), 3.87 (dd, *J* = 10.5, 7.0 Hz, 1H_c_), 4.22 (q, 2H, *J* = 6.9 Hz, CH_2_ of CH_2_CH_3_), 4.69 (dd, *J* = 10.5, 9.0 Hz, 1H_a_), 6.55–7.59 (m, 10H, Ar-H), 7.62 (s, 1H, CH=), 10.3 (s, 1H, D_2_O-exchangeable, NH). ^13^C-NMR (75 MHz, δ ppm, DMSO-*d*_6_); 13.9, 29.7, 37.8, 43.1, 46.9, 54.7, 56.3, 56.4, 56.8, 56.9, 59.0, 63.8, 76.7, 111.5, 113.7, 114.7, 115.4, 119.8,120.2, 121.5, 124.5, 126.3, 128.4, 128.6, 134.4, 136.0, 137.8, 139.7, 146.6, 148.9, 149.0, 149.8, 152.9, 154.5, 172.2, 204.1. MS *m/z* (%): 643 (M^+^ + 2, 8), 642 (M^+^ + 1, 39), 641 (M^+^, 70), 329 (10), 318 (9), 137 (4). Anal. For C_36_H_39_N_3_O_8_ (641.72): calcd. C, 67.38; H, 6.13; N, 6.55. Found: C, 67.31; H, 6.08; N, 6.48.

*Ethyl 1-N-methyl-spiro[2.3′]oxindole-spiro[3.3′′]5′′-(4-trifluro-methyl)benzylidene-1′′-N-carbo-xylate-4′′-piperidinone-4-(4-trifluro-methyl)phenyl-pyrrolidine* (**6i**). Green crystals, m.p. 136–137 °C (EtOH/Dioxane); IR: 3367 (NH), 1709, 1701, 1654 (3CO) cm^−1^. ^1^H-NMR (DMSO-*d*_6_): δ 1.15 (t, 3H, *J* = 7.2 Hz, CH_3_ of CH_2_CH_3_), 2.09 (s, 3H, NCH_3_), 3.24 (dd, *J* = 9.0, 7.0 Hz, 1H_b_), 3.32, 3.46 (2s, 4H, 2CH_2_of piperidinone ring), 3.82 (dd, *J* = 10.7, 7.0 Hz, 1H_c_), 4.23 (q, 2H, *J* = 7.2 Hz, CH_2_ of CH_2_CH_3_), 4.76 (dd, *J* = 10.7, 9.0 Hz, 1H_a_), 6.51–7.71 (m, 12H, Ar-H), 7.75 (s, 1H, CH=), 10.39 (s, 1H, D_2_O-exchangeable, NH). MS *m/z* (%): 659 (M^+^ + 2, 7), 658 (M^+^ + 1, 39), 657 (M^+^, 90), 344 (12), 339 (20), 145 (8). Anal. For C_34_H_29_F_6_N_3_O_4_ (657.61): calcd. C, 62.1; H, 4.44; N, 6.39.Found: C, 62.13; H, 4.36; N, 6. 37.

*Ethyl 1-N-methyl-spiro[2.3′]oxindole-spiro[3.3′′]5′′-(3,4,5-trimethoxy)benzylidene-1′′-N-carboxylate-4′′-piperidinone-4-(3,4,5-trimethoxy)phenyl-pyrrolidine* (**6j**). Yellow crystals, m.p. 115–116 °C (EtOH/Dioxane); IR: 3309 (NH), 1715, 1696, 1655 (3CO) cm^−1^. ^1^H-NMR (DMSO-*d*_6_): δ 1.20 (t, 3H, *J* = 7 Hz, CH_3_ of CH_2_CH_3_), 2.12 (s, 3H, NCH_3_), 3.26 (dd, *J* = 9.0, 7.0 Hz, 1H_b_), 3.27, 3.44 (2s, 4H, 2CH_2_ of piperidinone ring), 3.69, 3.71 (2s, 6H, 2OCH_3_), 3.74, 3.75 (2s, 6H, 2OCH3), 3.80, 3.82 (2s, 6H, 2OCH_3_), 3.87 (dd, *J* = 10.7, 7.0 Hz, 1H_c_), 4.23 (q, 2H, *J* = 7 Hz, CH_2_ of CH_2_CH_3_), 4.75 (dd, *J* = 10.7, 9.0 Hz, 1H_a_), 6.43–6.81 (m, 8H, Ar-H), 7.25 (s, 1H, CH=), 9.88 (s, 1H, D_2_O-exchangeable, NH). MS *m/z* (%): 703 (M^+^ + 2, 9), 702 (M^+^ + 1, 43), 701 (M^+^, 92), 364 (12), 368 (19), 339 (20) 167 (7). Anal. For C_38_H_43_N_3_O_10_ (701.77): calcd. C, 65.04; H, 6.18; N, 5.99. Found C, 64.95; H, 6.11; N, 5.92.

### 3.4. General Synthesis of Dispiropyrrolizine Oxindole Derivatives *(**8a**–**j**)*

A reaction mixture of isatin **1** (1.47 g, 1.0 mmol), l-proline **7** (1.15 g, 10 mmol), and ethyl 3,5-*bis*(aryl-methylidene)-*N*-carboxyl-4-piperidinone **4a**–**j** (1.0 mmol), was producedby refluxing it in methanol for 120 min at 60 °C, and was then poured on water. The solid that formed was collected and crystallized from suitable solvent to get white to green crystals of compounds **8a**–**j**.

*Ethyl spiro[3.3′′]-oxindole-spiro[2.3′]1'-carboxylate-5'-phenylmethylidene-tetra-hydro-4'(1H)-piperidinone-1-phenyl-hexahydro-1H-pyrrolizine* (**8a**). Yellow crystals, m.p. 127–128 °C (EtOH/Dioxane); IR: 3251 (NH), 1720, 1690, 1600 (3CO) cm^−1^. ^1^H-NMR (DMSO-*d*_6_): δ 1.15 (t, 3H, *J* = 6.9 Hz, CH_3_ of CH_2_CH_3_), 1.78–1.90 (m, 4H, 2CH_2_ of pyrrolizline), 2.40–2.48 (m, 2H, CH_2_ of pyrrolizine), 2.51–2.55 (m, 1H_b_), 3.26 (d, *J* = 10.1 Hz, 1H_a_), 3.53, 3.77 (2s, 4H, 2CH_2_ of piperidinone ring), 4.11 (q, 2H, *J* = 6.9 Hz, CH_2_ of CH_2_CH_3_), 6.68-7.53 (m, 14H, Ar-H), 7.64 (s, 1H, CH=), 8.53 (br, 1H, D_2_O-exchangeable, NH).^13^C-NMR (DMSO-*d*_6_): δ 13.9, 24.2, 28.5, 51.6, 32.7, 42.9, 46.6, 59.2, 61.1, 62.1, 71.4, 121.5, 124.5, 126.2, 126.3, 126.6, 128.2, 128.3, 128.4, 128.5, 128.7, 135.7, 136.1, 137.5, 139.4, 139.5, 152.9, 154.5, 173.2, 199.2. MS *m/z* (%): 549 (M^+^ + 2, 5), 548 (M^+^ + 1, 37), 547 (M^+^, 65), 470 (3), 457 (9), 412 (5), 302 (34), 257 (30), 206 (10), 158 (9), 97 (8), 77 (3). Anal. For C_34_H_33_N_3_O_4_ (547.65): calcd. C, 74.57; H, 6.07; N, 7.67. Found C, 74.33; H, 6.1; N, 7.6.

*Ethyl spiro[3.3′′]-oxindole-spiro[2.3′]1'-carboxylate-5'-(4-nitro)phenylmethylidene-tetrahydro-4'(1H)-piperidinone-1-(4-nitro)phenyl-hexahydro-1H-pyrrolizine* (**8b**). Yellow crystals, m.p. 160–161 °C (Dioxane); IR: 3255 (NH), 1701, 1692, 1620 (3CO) cm^−1^. ^1^H-NMR (DMSO-*d*_6_): δ 1.15 (t, 3H, *J* = 7 Hz, CH_3_ of CH_2_CH_3_), 1.78–1.98 (m, 4H, 2CH_2_ of pyrrolizline), 2.32–2.38 (m, 2H, CH_2_ of pyrrolizine), 2.71–2.75 (m, 1H_b_), 3.28 (d, *J* = 10.3 Hz, 1H_a_), 3.52, 3.71 (2s, 4H, 2CH_2_ of piperidinone ring), 4.21 (q, 2H, *J* = 7 Hz CH_2_ of CH_2_CH_3_), 6.76–7.52 (m, 12H, Ar-H), 7.66 (s, 1H, CH=), 8.62 (br, 1H, D_2_O-exchangeable, NH). ^13^C-NMR (DMSO-*d*_6_): δ 13.9, 23.7, 29.5, 31.6, 42.5, 46.7, 50.6, 59.1, 60.9, 61.5, 71.2, 120.9, 121.1, 121.4, 124.3, 126.5, 127.4, 128.3, 129.2, 136.0, 137.8, 139.7, 141.3,145.3, 145.7, 147.7, 152.7, 154.6, 172.6, 203.2. MS *m/z* (%): 639 (M^+^ + 2, 10), 638 (M^+^ + 1, 35), 637 (M^+^, 89), 503 (9), 494 (4), 242 (12), 205 (9), 122 (5), 97 (10). Anal. For C_34_H_31_N_5_O_8_ (637.65): calcd. C, 64.04; H, 4.90; N, 10.98. Found: C, 63.96; H, 4.84; N, 10.86.

*Ethyl spiro[3.3′′]-oxindole-spiro[2.3′]1'-carboxylate-5'-(4-chloro)phenylmethylidene-tetrahydro-4'(1H)-piperidinone-1-(4-chloro)phenyl-hexahydro-1H-pyrrolizine* (**8c**). Yellow crystals, m.p. 128–129 °C (EtOH/Dioxane); IR: 3251 (NH), 1701, 1678, 1616 (3CO) cm^−1^. ^1^H-NMR (DMSO-*d*_6_): δ 1.15 (t, 3H, *J* = 7.4 Hz, CH_3_ of CH_2_CH_3_), 1.78–1.89 (m, 4H, 2CH_2_of pyrrolizline), 2.33–2.38 (m, 2H, CH_2_ of pyrrolizine), 2.51–2.56 (m, 1H_b_), 3.35 (d, *J* = 10.4 Hz, 1H_a_), 3.78, 3.84 (2s, 4H, 2CH_2_ of piperidinone ring), 4.39 (q, 2H, *J* = 7.4 Hz, CH_2_ of CH_2_CH_3_), 6.72–7.49 (m, 12H, Ar-H), 7.56 (s, 1H, CH=), 8.48 (s, 1H, D_2_O-exchangeable, NH). ^13^C-NMR (DMSO-*d*_6_): δ 13.7, 23.8, 29.2, 32.0, 42.5, 46.0, 51.5, 59.0, 60.8, 61.8, 71.1, 121.4, 124.3, 126.7, 127.8, 128.0, 128.6, 128.9, 129.6, 131.6, 133.4, 133.7, 136.3, 137.4, 137.6, 139.8, 152.7, 154.4, 172.5, 200.2. MS *m/z* (%): 617 (M^+^ + 2, 67), 616 (M^+^ + 1, 59), 615 (M^+^, 80), 494 (7), 482 (14), 337 (20), 292 (15), 219 (8), 206 (20), 97 (10), 57 (6). Anal. For C_34_H_31_Cl_2_N_3_O_4_ (616.54): calcd. C, 66.24; H, 5.07; N, 6.82. Found: C, 66.13; H, 5.02; N, 6.80.

*Ethyl spiro[3.3′′]-oxindole-spiro[2.3′]1'-carboxylate-5'-(4-fluoro)phenylmethylidene-tetrahydro-4'(1H)-piperidinone-1-(4-fluoro)phenyl-hexahydro-1H-pyrrolizine* (**8d**). White crystals, m.p. 185–187 °C (Dioxane); IR: 3394 (NH), 1715, 1692, 1620 (3CO) cm^−1^. ^1^H-NMR (DMSO-*d*_6_): δ 1.18 (t, 3H, *J* = 7.2 Hz, CH_3_ of CH_2_CH_3_),1.75–1.96 (m, 4H, 2CH_2_ of pyrrolizline), 2.49–2.55 (m, 2H, CH_2_ of pyrrolizine), 2.51–2.57 (m, 1H_b_), 3.26 (d, *J* = 10.2 Hz, 1H_a_), 3.46, 3.77 (2s, 4H, 2CH_2_ of piperidinone ring), 4.24 (q, 2H, *J* = 7.2 Hz, CH_2_ of CH_2_CH_3_), 6.79–7.35 (m, 12H, Ar-H), 7.36 (s, 1H, CH=), 8.35 (s, 1H, D_2_O-exchangeable, NH). ^13^C-NMR (DMSO-*d*_6_): δ 13.7, 23.9, 28.9, 32.7, 42.4, 46.8, 50.8, 59.0, 60.8, 61.6, 71.2, 115.2, 115.3, 121.3, 124.2, 126.0, 128.2, 128.2, 129.9, 130.8,134.9, 136.5, 137.3, 139.7, 152.8, 154.4, 160.3, 162.2,173.1, 199.9. MS *m/z* (%): 585 (M^+^ + 2, 7), 584 (M^+^ + 1, 37), 583 (M^+^, 86), 206 (10), 97 (10). Anal. For C_34_H_31_F_2_N_3_O_4_ (583.64): calcd. C, 69.97; H, 5.35; N, 7.20. Found: C, 69.90; H, 5.28; N, 7.15.

*Ethyl spiro[3.3′′]-oxindole-spiro[2.3′]1'-carboxylate-5'-(4-methoxy)phenylmethylidene-tetrahydro-4'(1H)-piperidinone-1-(4-methoxy)phenyl-hexahydro-1H-pyrrolizine* (**8e**). Yellow crystals, m.p. 158–160 °C (EtOH/Dioxane); IR: 3421 (NH), 1715, 1662, 1600 (3CO) cm^−1^. ^1^H-NMR (DMSO-*d*_6_): δ 1.14 (t, 3H, *J* = 6.9 Hz, CH_3_ of CH_2_CH_3_), 1.78–1.96 (m, 4H, 2CH_2_ of pyrrolizline), 2.44–2.50 (m, 2H, CH_2_ of pyrrolizine), 2.59–2.65 (m, 1H_b_), 3.25 (d, *J* = 10.2 Hz, 1H_a_), 3.68, 3.82 (2s, 4H, 2CH_2_ of piperidinone ring), 3.69,3.75 (2s, 6H, 2OCH_3_), 4.25 (q, 2H, *J* = 6.9 Hz, CH_2_ of CH_2_CH_3_), 7.05–7.52 (m, 12H, Ar-H), 7.65 (s, 1H, CH=), 8.8 (s, 1H, D_2_O- exchangeable, NH). ^13^C-NMR (DMSO-*d*_6_): δ 13.8, 24.1, 29.1, 32.5 , 42.5, 46.7, 50.6, 56.1, 58.8, 58.9, 60.8, 61.6, 71.2, 114.2, 114.3, 121.5, 124.1, 126.9, 127.5, 127.7, 128.2, 129.2, 131.7, 136.3, 137.2, 139.6, 152.7, 154.2, 157.9, 159.9, 173.0, 202.2. MS *m/z* (%): 609 (M^+^ + 2, 8), 608 (M^+^ + 1, 97), 607 (M^+^, 45), 333 (20), 288 (30), 215 (15), 120 (8). Anal. For C_36_H_37_N_3_O_6_ (607.71): calcd. C, 71.15; H, 6.14; N, 6.91. Found: C, 71.13; H, 6.12; N, 6.87.

*Ethyl spiro[3.3′′]-oxindole-spiro[2.3′]1'-carboxylate-5'-(3,4-dichloro)phenylmethylidene-tetrahydro-4'(1H)-piperidinone-1-(3,4-dichloro)phenyl-hexahydro-1H-pyrrolizine* (**8f**). White crystals, m.p. 158–159 °C (EtOH/Dioxane); IR: 3255 (NH), 1701, 1681, 1620 (3CO) cm^−1^. ^1^H-NMR (DMSO-*d*_6_): δ 1.16 (t, 3H, *J* = 7.2 Hz, CH_3_ of CH_2_CH_3_), 1.77–1.98 (m, 4H, 2CH_2_ of pyrrolizline), 2.46–2.52 (m, 2H, CH_2_ of pyrrolizine), 2.55–2.75 (m, 1H_b_), 3.26 (d, *J* = 10.2 Hz, 1H_a_), 3.34, 3.62 (2s, 4H, 2CH_2_ of piperidinone ring), 4.32 (q, 2H, *J* = 7.2 Hz, CH_2_ of CH_2_CH_3_), 6.79–7.35 (m, 10H, Ar-H), 7.38 (s, 1H, CH=), 8.45 (s, 1H, D_2_O-exchangeable, NH). MS *m/z* (%): 685 (M^+^, 49), 684 (100), 683 (78), 371 (21), 353 (14), 327 (14), 143 (8). Anal. For C_34_H_29_Cl_4_N_3_O_4_ (685.42) calcd. C, 59.58; H, 4.26; N, 6.13. Found: C, 59.56; H, 4.19; N, 6.07.

*Ethyl spiro[3.3′′]-oxindole-spiro[2.3′]1'-carboxylate-5'-(2,4-difluoro)phenylmethylidene-tetrahydro-4'(1H)-piperidinone-1-(2,4-difluoro)phenyl-hexahydro-1H-pyrroliz-ine* (**8g**). Pale green crystals, m.p. 132–133 °C (Methanol); IR: 3433 (NH), 1705, 1681, 1616 (3CO) cm^−1^. ^1^H-NMR (DMSO-*d*_6_): δ 1.13 (t, 3H, *J* = 7.4 Hz, CH_3_ of CH_2_CH_3_), 1.72–1.89 (m, 4H, 2CH_2_ of pyrrolizline), 2.42–2.49 (m, 2H, CH_2_ of pyrrolizine), 2.69–2.75 (m, 1H_b_), 3.30 (d, *J* = 10.0 Hz, 1H_a_), 3.45, 3.88 (2s, 4H, 2CH_2_ of piperidinone ring), 4.30 (q, 2H, *J* = 7.4 Hz, CH_2_ of CH_2_CH_3_), 6.71–7.61 (m, 10H, Ar-H), 7.66 (s, 1H, CH=), 8.48 (s, 1H, D_2_O-exchangeable, NH). MS *m/z* (%): 621 (M^+^ + 2, 7), 620 (M^+^ + 1, 38), 619 (M^+^, 55), 294 (23), 393 (9), 221 (4), 93 (4). Anal. for C_34_H_29_F_4_N_3_O_4_ (619.62): calcd. C, 65.91; H, 4.72; N, 6.78. Found: C, 65.88; H, 4.68; N, 6.73.

*Ethyl spiro[3.3′′]-oxindole-spiro[2.3′]1'-carboxylate-5'-(3,4-dimethoxy)phenylmethylidene-tetrahydro-4'(1H)-piperidinone-1-(3,4-dimethoxy)phenyl-hexahydro-1H-pyrro-lizine* (**8h**). Yellow crystals, m.p. 231–233 °C (EtOH/Dioxane); IR: 3421 (NH), 1700, 1697, 1616 (3CO) cm^−1^. ^1^H-NMR (DMSO-*d*_6_): δ 1.14 (t, 3H, *J* = 7.1 Hz, CH_3_ of CH_2_CH_3_), 1.78–2.02 (m, 4H, 2CH_2_ of pyrrolizline), 2.38–2.48 (m, 2H, CH_2_ of pyrrolizine), 2.59–2.73 (m, 1H_b_), 3.33 (d, *J* = 10 Hz, 1H_a_), 3.52, 3.65 (2s, 4H, 2CH_2_of piperidinone ring), 3.69, 3.71 (2s, 6H, 2OCH_3_), 3.79, 3.82 (2s, 6H, 2OCH_3_), 4.21 (q, 2H, *J* = 7.1 Hz, CH_2_ of CH_2_CH_3_), 6.79–7.65 (m, 10H, Ar-H), 7.72 (s, 1H, CH=), 8.31 (s, 1H, D_2_O-exchangeable, NH). ^13^C-NMR (DMSO-*d*_6_): δ 13.7, 23.6, 29.5, 31.9, 42.5, 46.6, 50.9, 56.2, 56.4, 56.7, 56.8, 59.5, 61.3, 61.4, 71.3, 111.7, 113.2, 115.2, 119.8, 121.6, 121.2, 124.7, 126.5, 128.4, 128.5, 132.6, 136.4, 137.3, 139.2, 147.2, 149.1, 149.7, 149.9, 152.9, 154.5, 173.0, 200.9. MS *m/z* (%): 669 (M^+^ + 2, 10), 668 (M^+^ + 1, 43), 667 (M^+^, 92), 362 (31), 318 (9), 243 (10), 90 (20), 93 (10). Anal. For C_38_H_41_N_3_O_8_ (667.76): calcd. C, 68.35; H, 6.19; N, 6.29. Found C, 68.28; H, 6.14; N, 6.24.

*Ethyl spiro[3.3′′]-oxindole-spiro[2.3′]1'-carboxylate-5'-(4-trifluromethyl)phenyl-methylidene-tetrahydro-4'(1H)-piperidinone-1-(4-trifluromethyl)phenyl-hexahydro-1H-pyrrolizine* (**8i**). Green crystals, m.p. 137–139 °C (EtOH/Dioxane); IR: 3371 (NH), 1701, 1690, 1616 (3CO) cm^−1^. ^1^H-NMR (DMSO-*d*_6_): δ 1.18 (t, 3H, *J* = 7 Hz, CH_3_ of CH_2_CH_3_), 1.76–1.98 (m, 4H, 2CH_2_ of pyrrolizline), 2.46–2.50 (m, 2H, CH_2_ of pyrrolizine), 2.59–2.74 (m, 1H_b_), 3.96 (d, *J* = 10.3 Hz, 1H_a_), 3.35, 3.77 (2s, 4H, 2CH_2_ of piperidinone ring), 4.32 (q, 2H, *J* = 7 Hz, CH_2_ of CH_2_CH_3_), 6.79–7.35 (m, 12H, Ar-H), 7.42 (s, 1H, CH=), 8.42 (s, 1H, D_2_O-exchangeable, NH).MS *m/z* (%): 685 (M^+^ + 2, 7), 684 (M^+^ + 1, 39), 683 (M^+^, 92), 370 (13), 325 (7), 251 (13), 145 (6). Anal. For C_36_H_31_F_6_N_3_O_4_ (683.65): calcd. C, 63.25; H, 4.57; N, 6.15. Found: C, 63.18; H, 4.51; N, 6.08.

*Ethyl spiro[3.3′′]-oxindole-spiro[2.3′]1'-carboxylate-5'-(3,4,5-trimethoxy)phenyl-methylidene-tetrahydro-4'(1H)-piperidinone-1-(3,4,5-trimethoxy)phenyl-hexahydro-1H-pyrrolizine* (**8j**). Yellow crystals, m.p. 123–124 °C (EtOH/Dioxane); IR: 3464 (NH), 1701, 1692, 1620 (3CO) cm^−1^. ^1^H-NMR (DMSO-*d*_6_): δ 1.15 (t, 3H, *J* = 6.9 Hz, CH_3_ of CH_2_CH_3_), 1.82–2.09 (m, 4H, 2CH_2_ of pyrrolizline), 2.44–2.50 (m, 2H, CH_2_ of pyrrolizine), 2.72–2.80 (m, 1H_b_), 3.25 (d, *J* = 10.1 Hz, 1H_a_), 3.46, 3.65 (2s, 4H, 2CH_2_ of piperidinone ring), 3.69, 3.71 (2s, 6H, 2OCH_3_), 3.74,3.76 (2s, 6H, 2OCH_3_), 3.82, 3.84 (2s, 6H, 2OCH_3_), 4.19 (q, 2H, *J* = 6.9 Hz, CH_2_ of CH_2_CH_3_), 6.39–7.55 (m, 8H, Ar-H), 7.62 (s, 1H, CH=), 8.37 (s, 1H, D_2_O-exchangeable, NH). MS *m/z* (%): 729 (M^+^ + 2, 2), 728 (M^+^ + 1, 10), 727 (M^+^, 42), 726 (94), 347 (19), 392 (25), 275 (17), 167 (9). Anal. For C_40_H_45_N_3_O_10_ (727.81): calcd. C, 66.01; H, 6.23; N, 5.77. Found: C, 65.94; H, 6.14; N, 5.65.

### 3.5. Microbiological Assay

An agar diffusion well method was used to determine the antimicrobial activity. The microorganism inoculums were uniformly spread using a sterile cotton swab on a sterile Petri dish containing Malt extract agar (for fungi) and nutrient agar (for bacteria). Each sample (100 μL) was added to each well (6 mm diameter holes cut in the agar gel, 20 mm apart from one another). The systems were incubated for 24–48 h at 37 °C (for bacteria) and at 28 °C (for fungi). After incubation, microorganism growth was observed. The inhibition of bacterial and fungal growth was measured in mm. Tests were performed in triplicate [26]. 

## 4. Conclusions

In conclusion, we synthesized new derivatives of spirooxindole-spiropiperidinone- pyrrolidinesnd-spirooxindole-spiropiperidinone-pyrrolizinesthrougha 1,3-dipolar cycloaddition reaction of azomethineylides, generated from isatin, sarcosine, and l-proline through a decarboxylative route with dipolarophile **4a**–**j**. All of the newly synthesized compounds were evaluated for their antimicrobial activities and the minimum inhibitory concentration (MIC) of the most active compounds against the test organisms. Four compounds from the series have emerged as potent antibacterial and antifungal agents.

## Figures and Tables

**Table 1:** Optimization of reaction condition.

Entry	Solvent	Temp (°C)	Yield ^a^ (%)
1	THF	60	54
2	Toluene	60	trace
3	CH_3_CN	60	72
4	CH_3_OH	60	92
5	EtOH	60	85
6	Water	60	NR
7	CH_3_OH	30	58
8	CH_3_OH	45	66

^a^ isolated yield based on isatin.

**Table 2:** Yields of spiropyrrolidine and spiropyrrolizinebisoxindoles.

Entry	Ar	6 & 8	Yield ^a^ (%)
1	C_6_H_5_	**6a**	92
2	4-NO_2_C_6_H_4_	**6b**	95
3	4-ClC_6_H_4_	**6c**	91
4	4-FC_6_H_4_	**6d**	92
5	4-OCH_3_C_6_H_4_	**6e**	84
6	3,4-Cl_2_C_6_H_3_	**6f**	87
7	2,4-F_2_C_6_H_3_	**6g**	89
8	3,4-OCH_3_·C_6_H_3_	**6h**	79
9	4-F_3_CC_6_H_4_	**6i**	86
10	3,4,5-OCH_3_ C_6_H_2_	**6j**	81
11	C_6_H_5_	**8a**	90
12	4-NO_2_C_6_H_4_	**8b**	88
13	4-ClC_6_H_4_	**8c**	82
14	4-FC_6_H_4_	**8d**	85
15	4-OCH_3_C_6_H_4_	**8e**	80
16	3,4-Cl_2_C_6_H_3_	**8f**	82
17	2,4-F_2_C_6_H_3_	**8g**	83
18	3,4-OCH_3_·C_6_H_3_	**8h**	81
19	4-F_3_CC_6_H_4_	**8i**	79
20	3,4,5-OCH_3_ C_6_H_2_	**8j**	87

^a^ isolated yield based on isatin.

**Table 3:** Antimicrobial activity of the synthesized compounds.

Compd. No.	Gram Positive Bacteria		Gram Negative Bacteria		Fungi			
	*S. pneumoniae*	*B. subtilis*	*P. aeruginosa*	*E. coli*	*A. fumigatus*	*S. racemosum*	*G. candidum*	*C. albicans*
**6b**	42.34 ± 0.15	74.25 ± 0.42	NA	49.25 ± 0.53	42.36 ± 0.33	49.32 ± 0.25	63.25 ± 0.34	NA
**6c**	96.25 ± 0.2	97.24 ± 0.3	49.25 ± 0.1	74.29 ± 0.3	96.25 ± 0.1	74.25 ± 0.2	95.36 ± 0.2	91.29± 0.1
**6e**	53.21 ± 0.43	63.28 ± 0.53	NA	24.29 ± 0.25	53.24 ± 0.58	63.29 ± 0.25	74.16 ± 0.38	NA
**6g**	49.21 ± 0.44	53.21 ± 0.67	NA	21.34 ± 0.46	25.36 ± 0.25	49.24 ± 0.34	49.24 ± 0.58	NA
**6i**	74.12 ± 0.63	74.95 ± 0.32	NA	42.34 ± 0.46	53.21 ± 0.36	63.24 ± 0.44	78.21 ± 0.58	NA
**6j**	63.24 ± 0.63	74.21 ± 0.32	NA	34.25 ± 0.46	42.15 ± 0.36	49.21 ± 0.44	53.21 ± 0.58	NA
**8b**	74.52 ± 0.44	91.25 ± 0.63	NA	49.25 ± 0.25	74.32 ± 0.39	82.63 ± 0.16	90.33 ± 0.58	NA
**8c**	34.25 ± 0.44	42.67 ± 0.25	NA	22.14 ± 0.33	39.25 ± 0.25	39.46 ± 0.58	42.67 ± 0.17	NA
**8e**	78.26 ± 0.34	96.25 ± 0.25	NA	49.25 ± 0.58	74.25 ± 0.63	78.23 ± 0.27	82.34 ± 0.35	NA
**8g**	95.44 ± 0.44	95.34 ± 0.58	22.14 ± 0.58	74.21 ± 0.19	53.22 ± 0.34	74.25 ± 0.25	92.68 ± 0.58	NA
**8i**	74.25 ± 0.44	96.25 ± 0.63	NA	34.24 ± 0.25	74.25 ± 0.39	49.25 ± 0.58	78.34 ± 0.58	NA
**8j**	74.65 ± 0.43	78.34 ± 0.53	NA	42.37 ± 0.33	82.65 ± 0.25	78.34 ± 0.25	98.25 ± 0.38	NA
Ampicillin	96.25 ± 0.2	97.24 ± 0.3	NA	NA	NA	NA	NA	NA
Gentamicin	NA	NA	49.25±0.1	74.29 ± 0.3	NA	NA	NA	NA
Amphotericin B	NA	NA	NA	NA	96.25 ± 0.1	74.25 ± 0.2	95.36 ± 0.2	91.29 ± 0.1

NA: No activity.

**Table 4:** Antimicrobial Activity as MICS (µg/mL) of tested samples against tested microorganisms.

Compd. No.	Gram Positive Bacteria		Gram Negative Bacteria		Fungi			
	*S. pneumoniae*	*B. subtilis*	*P. aeruginosa*	*E. coli*	*A. fumigatus*	*S. racemosum*	*G. candidum*	*C. albicans*
**6c**	0.12	0.015	15.63	1.95	0.015	1.95	0.015	0.06
**8b**	1.95	0.06	NA	15.63	1.95	0.49	0.12	NA
**8e**	0.98	0.015	NA	15.63	1.95	0.98	0.49	NA
**8g**	0.015	0.015	NA	1.95	7.81	1.95	0.03	NA
Amphotericin B	NA	NA	NA	NA	**0.015**	**1.95**	**0.015**	**0.06**
*A*mpicillin	**0.015**	**0.007**	NA	NA	NA	NA	NA	NA
*Gentamicin*	NA	NA	**15.63**	**1.95**	NA	NA	NA	NA

NA: No activity.

**Table 5:** Antimicrobial Activity as IC_50_ (µg/mL) of tested samples against tested microorganisms.

Compd. No.	Gram Positive Bacteria		Gram Negative Bacteria		Fungi			
	*S. pneumoniae*	*B. subtilis*	*P. aeruginosa*	*E. coli*	*A. fumigatus*	*S. racemosum*	*G. candidum*	*C. albicans*
**6c**	3.98	1.34	36.25	17.63	1.96	16.37	1.45	2.16
**8b**	16.35	2.92	NA	32.84	18.36	10.64	4.29	NA
**8e**	12.36	1.36	NA	32.12	16.32	11.32	9.25	NA
**8g**	1.73	1.24	>125	15.98	21.36	16.38	2.09	NA
Amphotericin B	NA	NA	NA	NA	**1.25**	**14.27**	**1.16**	**2.48**
*A*mpicillin	**1.53**	**1.06**	NA	NA	NA	NA	NA	NA
*Gentamicin*	NA	NA	**20.31**	**16.62**	NA	NA	NA	NA

NA: No activity.
